# microRNAs Regulate Cell-to-Cell Variability of Endogenous Target Gene Expression in Developing Mouse Thymocytes

**DOI:** 10.1371/journal.pgen.1005020

**Published:** 2015-02-25

**Authors:** Rory Blevins, Ludovica Bruno, Thomas Carroll, James Elliott, Antoine Marcais, Christina Loh, Arnulf Hertweck, Azra Krek, Nikolaus Rajewsky, Chang-Zheng Chen, Amanda G. Fisher, Matthias Merkenschlager

**Affiliations:** 1 MRC Clinical Sciences Centre, Imperial College London, London, United Kingdom; 2 Department of Microbiology and Immunology and Baxter Laboratory for Stem Cell Biology, Stanford University School of Medicine, Stanford, California, United States of America; 3 Systems Biology of Gene Regulatory Elements, Max Delbrück Center Berlin, Berlin, Germany; University of California San Francisco, UNITED STATES

## Abstract

The development and homeostasis of multicellular organisms relies on gene regulation within individual constituent cells. Gene regulatory circuits that increase the robustness of gene expression frequently incorporate microRNAs as post-transcriptional regulators. Computational approaches, synthetic gene circuits and observations in model organisms predict that the co-regulation of microRNAs and their target mRNAs can reduce cell-to-cell variability in the expression of target genes. However, whether microRNAs directly regulate variability of endogenous gene expression remains to be tested in mammalian cells. Here we use quantitative flow cytometry to show that microRNAs impact on cell-to-cell variability of protein expression in developing mouse thymocytes. We find two distinct mechanisms that control variation in the activation-induced expression of the microRNA target *CD69*. First, the expression of miR-17 and miR-20a, two members of the miR-17-92 cluster, is co-regulated with the target mRNA *Cd69* to form an activation-induced incoherent feed-forward loop. Another microRNA, miR-181a, acts at least in part upstream of the target mRNA Cd69 to modulate cellular responses to activation. The ability of microRNAs to render gene expression more uniform across mammalian cell populations may be important for normal development and for disease.

## Author Summary

microRNAs are integral to many developmental processes and may 'canalise' development by reducing cell-to-cell variation in gene expression. This idea is supported by computational studies that have modeled the impact of microRNAs on the expression of their targets and the construction of artificial incoherent feedforward loops using synthetic biology tools. Here we show that this interesting principle of microRNA regulation actually occurs in a mammalian developmental system. We examine cell-to-cell variation of protein expression in developing mouse thymocytes by quantitative flow cytometry and find that the absence of microRNAs results in increased cell-to-cell variation in the expression of the microRNA target *Cd69*. Mechanistically, T cell receptor signaling induces both *Cd69* and miR-17 and miR-20a, two microRNAs that target *Cd69*. Co-regulation of microRNAs and their target mRNA dampens the expression of *Cd69* and forms an incoherent feedforward loop that reduces cell-to-cell variation on CD69 expression. In addition, miR-181, which also targets *Cd69* and is a known modulator of T cell receptor signaling, also affects cell-to-cell variation of CD69 expression. The ability of microRNAs to control the uniformity of gene expression across mammalian cell populations may be important for normal development and for disease.

## Introduction

The complexity of developmental processes in metazoans relies on mechanisms that confer a degree of robustness against environmental and genetic variation [1]. microRNAs are small non-coding RNAs that negatively regulate gene expression at the post-transcriptional level by reducing mRNA stability and/or translation. Their role in dampening gene expression makes microRNAs potential building blocks for gene regulatory circuits that can stabilize gene regulatory networks [2–5].

Gene expression is subject to intrinsic stochasticity associated with mRNA transcription and translation, as well as extrinsic noise such as fluctuations in upstream regulators. Gene expression noise is not restricted to protein coding genes: the expression of primary microRNA transcripts, their processing into pre-microRNAs, nuclear export, processing into mature microRNAs, association with RISC components, etc., presumably all have stochastic components. The participation of microRNAs in the regulation of protein-coding genes could therefore add noise contained in both microRNA and protein-coding systems. Feed-forward loops (FFLs) are recurrent network motifs that can reduce gene expression noise by buffering fluctuations in upstream regulators [6]. Placing the expression of a microRNA and its target mRNA under the control of common upstream regulators can link the production of mRNAs to the production of microRNAs that target the mRNAs. Theoretical considerations [2] and computational simulations [7, 8] suggest that this circuit topology, which resembles an incoherent FFL, allows microRNAs to buffer protein expression against fluctuations in the activity of upstream regulators [9]. *In silico* models predict that FFL regulation enables microRNAs to reduce not only the level of target gene expression, but also cell-to-cell variability [7, 8]. Data from synthetic circuits indicate that co-expression of microRNAs and target mRNAs can reduce temporal fluctuations and in some cases cell-to-cell variability in reporter gene expression [7, 10].

Emerging experimental evidence supports a role for microRNAs in biological robustness [2]. microRNAs affect several phenotypic traits in *Drosophila*, for example by stabilizing the regulation of the enhancer of split transcription factor to guide sensory organ development under conditions of environmental flux [11]. Loss of microRNAs can increase the variation of primordial germ cell numbers [12, 13] and sensory bristles [14], and quantitative phenotypic traits in the *Drosophila* cuticle [15]. These data demonstrate that microRNAs can buffer variation in phenotypic traits, but it is not clear whether this is achieved by reduced variation in the expression of microRNA target genes or the operation of thresholds for phenotypic outcomes [16]. Zebrafish miR-26b and *ctdsp2* mRNA are encoded by the same primary transcript, and *ctdsp2* mRNA is a target of miR-26b [17]. The processing of miR-26b is developmentally controlled during neuronal differentiation, effectively initiating a microRNA-mediated incoherent FFL but the consequences for cell-to-cell variation in the expression of *ctdsp2* have yet to be established [17]. microRNAs can dampen temporal oscillations in gene expression in *C. elegans* [18] and reduce fluctuations in the average expression of reporter constructs in mammalian cells [19]. Measurements at the population level, but not in individual cells, showed that methyl CpG-binding protein 2 (MeCP2) acts through BDNF to induce the neuronal miRNA miR-132, which feeds back to repress MeCP2 [20]. However, simple negative feedback loops like this may increase noise as determined experimentally and computationally [7]. The miR-17-92 family forms a complex network with Cyclin D1 in neuronal progenitors, and the variability of Cyclin D1 expression was increased by heterozygosity in *Dicer* [21]. The relationship between microRNAs and variability of target gene expression is complicated in this system, since miR-17-92 is required for the differentiation of mouse cortical neuronal progenitors [22], and reduced microRNA expression affects the frequency of proliferating neuronal progenitors as well as the expression of *Ccnd1* within them [21, 22]. That loss of microRNAs can also result in reduced variability in the expression of pluripotency markers was recently demonstrated for mouse ES cells [23].

Here we address the impact of the microRNA biogenesis pathway on cell-to-cell variability of endogenous gene expression in mouse thymocytes (developing T cells). This system offers a number of key advantages. T cell development proceeds in a series of discrete developmental steps that are defined by the expression of cell surface markers [24]. This allows for (i) the precise definition and isolation of cell populations at specific developmental stages for the molecular characterization of microRNA and target mRNA expression, (ii) the use of developmentally regulated Cre transgenes for the synchronous deletion of conditional alleles of the RNase III enzyme Dicer, (iii) the verification of reduced microRNA expression at defined developmental stages, and (vi) like-for-like comparisons between control and Dicer-deficient cells at the same developmental stage. Thymocytes readily form cell suspensions that are ideally suited for analysis and sorting by flow cytometry, and high-quality reagents are available to enable quantitative flow cytometry at the single cell level [25]. Using this approach we demonstrate that microRNAs can reduce cell-to-cell variation of target gene expression in mammalian cells. The activation-induced microRNA target CD69 was regulated by microRNAs in two different ways. miR-181a affected variation by modulating the responsiveness of thymocytes to activation signals, acting at least in part upstream on the target mRNA *Cd69*. Members of the miR-17-92 cluster were co-regulated with the target mRNA *Cd69*, resembling an activation-induced incoherent FFL.

## Results

### microRNA-dependent regulation of gene expression in developing thymocytes

**Fig 1:**
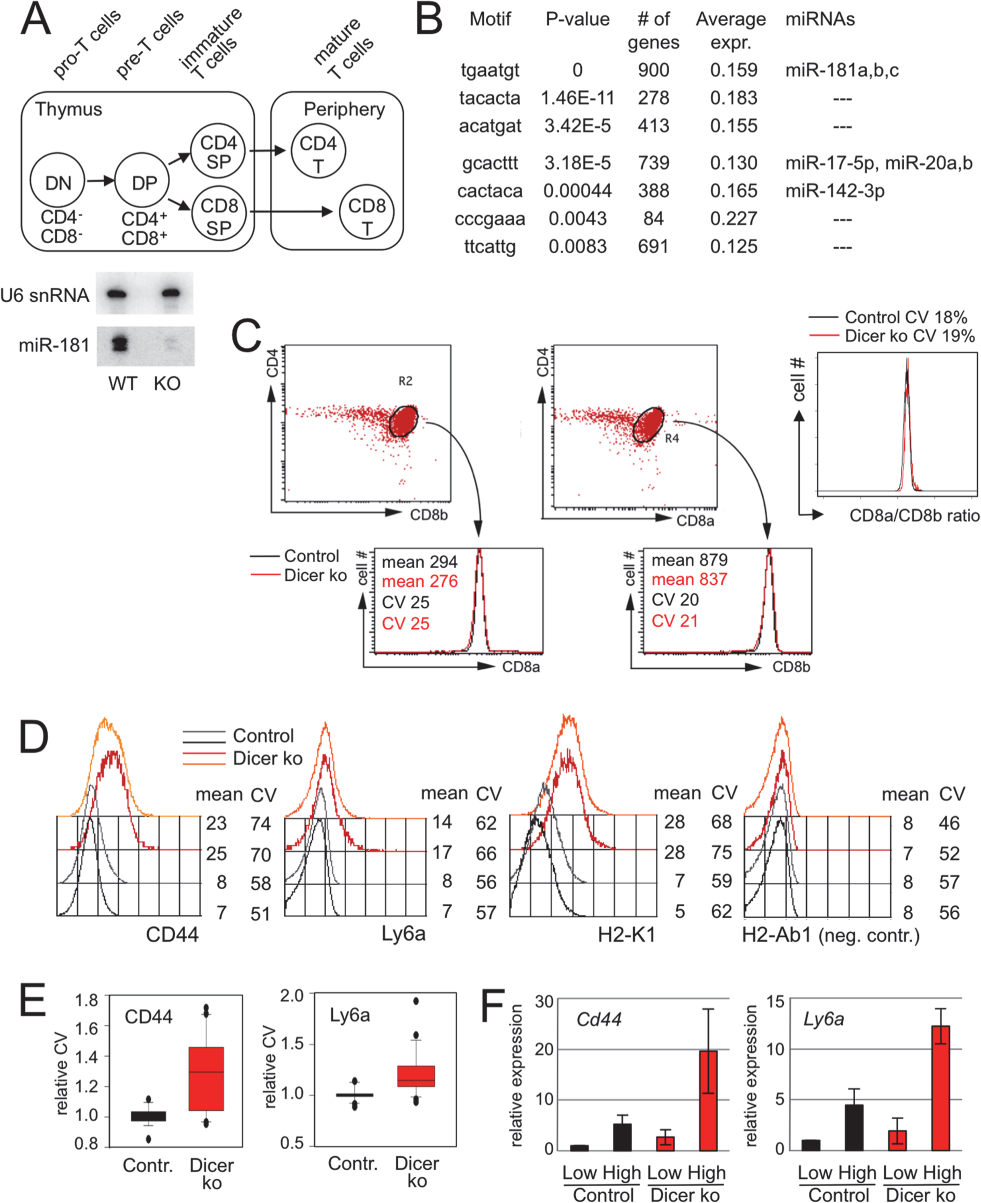
microRNA-dependent regulation of gene expression in developing thymocytes. A) Schematic of T cell differentiation. Abbreviations are DN: double negative for CD4 and CD8; DP: double positive for CD4 and CD8; Periphery: peripheral lymphoid organs. The expression of mature microRNAs, including miR-181a, is reduced by ∼ 90% at the DP stage of development as demonstrated by northern blotting with U6 snRNA as a loading control, data from [26]. B) 3'UTR motifs and microRNAs associated with positive fold-change of transcript levels in Dicer-deficient DP thymocytes as determined by miReduce [27]. 'Average expr.' denotes the fold-change in the expression of mRNAs with the indicated 3'UTR motifs between Dicer-deficient and control DP thymocytes. C) Control and Dicer-deficient DP thymocytes as defined by staining for CD4 versus CD8a (left) and CD4 versus CD8b (middle). Both the mean expression and the CV of CD8a and CD8b are comparable between control and Dicer-deficient DP thymocytes. The ratio of CD8a/CD8b expression was calculated for individual control (left) and Dicer-deficient (middle) DP thymocytes. The CV of these ratios represents experimental noise (right) [25]. D) CD44, Ly6a and H2-Kb are examples of proteins encoded by transcripts that are deregulated in Dicer-deficient DP thymocytes. The panels show representative flow cytometry histograms of CD44, Ly6a and H2-K1 expression by Dicer^lox/lox^ (black/grey) and Dicer^Δ/Δ^ (red/orange) DP thymocytes gated on low levels of T cell receptor (TCR) expression. The MHC class II antigen H-2Ab1 is not expressed by mouse T cells and served as a negative control. Numbers indicate the mean expression level and the coefficient of variation (CV). Representative of 3–5 biological replicates. E) Analysis of 10–30 biological replicates showed an increase in the CV of CD44 protein expression of 30% and an increase in the CV of Ly6a protein expression of 15% in Dicer-deficient versus control DP thymocytes. F) DP thymocytes were sorted by flow cytometry according to the level of CD44 and Ly6a expression by individual cells. Analysis of the sorted populations by quantitative RT-PCR indicates that protein expression as assessed by flow cytometry predicts mRNA expression.

We previously characterised an experimental system where a developmentally regulated *Lck*-Cre transgene deletes a conditional *Dicer* allele in developing mouse thymocytes [26]. As a result, the expression of Dicer-dependent microRNAs was reduced by ∼90% at the CD4 CD8 double positive (DP) stage of development (Fig. 1A) [26]. miReduce analysis [27] of 3'UTR motifs associated with post-transcriptional de-repression in *Lck*-Cre DP thymocytes (see GSE57511) showed enrichment for microRNAs miR-181, miR-17 and miR-142 (Fig. 1B).

We evaluated flow cytometry as an approach to determine protein expression by individual cells. To estimate technical noise we examined CD8a and CD8b, which are expressed as obligate heterodimers in DP thymocytes. The mean expression and the coefficient of variation (CV) of CD8a and CD8b were very similar for control and Dicer-deficient DP thymocytes (Fig. 1C, left and centre), as was the ratio of CD8a/CD8b expression for individual control and Dicer-deficient DP thymocytes (Fig. 1D, right). The CV of these ratios defines the upper bound of technical noise [25]. Based on published criteria for quantitative flow cytometry [25] we identified antibodies directed against putative microRNA targets including CD44, a predicted target of miR-21 (www.targetscan.org) and established target of miR-34 [28] as well as the predicted miR-181 targets Ly6a and H2-K1 (www.targetscan.org; Fig. 1D). As expected based on elevated mRNA expression (see GSE57511), Dicer-deficient thymocytes showed higher average expression of CD44, Ly6a and H2-K1 than control cells. Interestingly, the cell-to-cell variation of CD44, Ly6a and H2-K1 expression was also increased in Dicer-deficient thymocytes (Fig. 1D, E) while there was no change in the negative staining control, MHC class II (H2-Ab1; Fig. 1D).

We used the CV as a stringent measure of variation because in contrast to the standard deviation (SD), the CV is expected to decrease as the mean expression increases (CV = standard deviation/mean). An increase in the CV at the same time as an increase in mean protein expression therefore unambiguously indicates an increase in cell-to-cell variation (Fig. 1D, E). As the CV is expected to decrease with the level of expression, the finding of an increased CV in Dicer-deficient cells that expressed higher protein levels prompted several control experiments. First, we asked whether the increased CV was explained by residual microRNA-retaining cells. Experimental mixing and computational modeling experiments indicated that this was highly unlikely (S1 Fig.). Second, the apparent impact on the CV could be due to technical limitations in the detection of low levels of protein expression: if microRNAs reduce expression below the sensitivity or our instrumentation we would detect expression—and associated noise—only in Dicer-deficient cells but not in wild type cells. To address this concern we asked whether the level of protein expression detected in wild type and Dicer-deficient cells was biologically meaningful. We sorted control and Dicer-deficient thymocytes according to the level of CD44 and Ly6a protein detected by flow cytometry and carried out quantitative reverse transcriptase (RT)-PCR for *Cd44* and *Ly6a* transcripts (Fig. 1F). The data correlated CD44 and Ly6a protein expression with the abundance of *Cd44* and *Ly6a* mRNA in both control and Dicer-deficient DP thymocytes. Although *Cd44, Ly6a* and *H2-K1* mRNAs are not confirmed direct microRNA targets in thymocytes, these data demonstrate that our instrumentation discriminates meaningful levels of protein expression.

### Dicer-deficient DP thymocytes show increased variation in inducible CD69 expression

**Fig 2:**
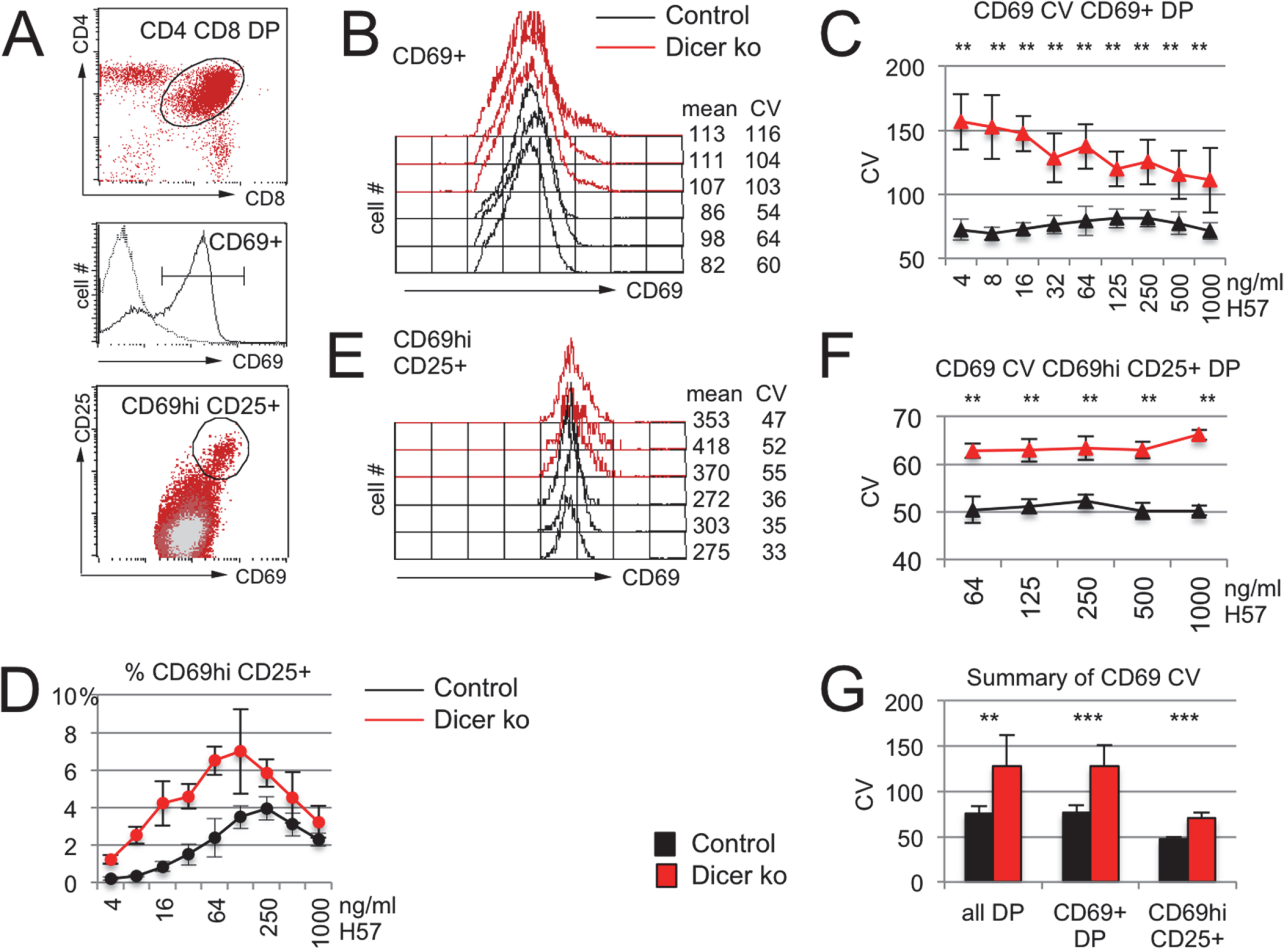
Increased CV of inducible CD69 expression in Dicer-deficient thymocytes. A) Activation of CD4+ CD8+ DP thymocytes (oval, top) results in CD69 expression (horizontal line defines CD69+ cells, middle) and definition of CD69hi cells (oval, bottom). B) Histogram overlays of CD69 expression by CD69+ DP thymocytes activated for 18 hours with 125ng H57/ml. Mean and CV of CD69 expression are indicated. C) The CV of CD69 expression is higher in Dicer-deficient than in control CD69+ DP thymocytes over a range of activation conditions (n = 4–7 per data point, ** P<0.001). See S1 Table for additional data. D) The frequency of CD69hi CD25+ DP thymocytes is higher in Dicer-deficient than in control DP thymocytes. E) Histogram overlays of CD69 expression by CD69hi CD25+ thymocytes activated for 18 hours with 125ng H57/ml. Mean and CV of CD69 expression are indicated. See S1 Table for additional data. F) The CV of CD69 expression is higher in Dicer-deficient than in control CD69hi CD25+ DP thymocytes (n = 4 per data point, ** P<0.001). See S1 Table for additional data. G) Summary of CD69 CV data for Dicer-deficient DP thymocytes, CD69+ DP thymocytes and CD69hi CD25+ DP thymocytes.

To unambiguously determine the impact of microRNAs on cell-to-cell variability of target gene expression on a direct microRNA target in thymocytes we focused on CD69, which is inducibly expressed in response to T-cell activation [29]. CD69 controls cell migration and sphingosine 1-phosphate signaling [30], and the *Cd69* mRNA is a well-characterised target of miR-181 and other microRNAs [31–33]. In response to activation signals through the T cell receptor (TCR), DP thymocytes initiated the expression of CD69 (Fig. 2A), and graded activation signals induced a proportional increase of *Cd69* mRNA and CD69 protein (S2 Fig.). As expected for an established microRNA target, average CD69 levels were higher in Dicer-deficient than in control DP thymocytes (S2B Fig.). In addition, Dicer-deficient DP thymocytes showed an increase in the CV of CD69 expression (Fig. 2B), and this increase was seen over a range of activation conditions (Fig. 2C). The broader distribution of CD69 expression among Dicer-deficient DP cells was due in part to a greater fraction of CD69^hi^ cells (Fig. 2D, characterized by the co-expression of CD25 Fig. 2A). In addition, Dicer-deficient DP thymocytes showed an increased CV of CD69 expression within the CD69^hi^ CD25^+^ subset (Fig. 2E, F). Hence, Dicer-deficient DP thymocytes showed increased cell-to-cell variability in the expression of the microRNA target CD69. This was true whether the CV was assessed for the entire DP thymocyte population, or separately for the CD69^+^ population or the CD69^high^ CD25^+^ subset (Fig. 2G).

Taken together, our results indicate that microRNAs can shape not only the level but also the cell-to-cell variability of protein expression in developing thymocytes. To investigate the underlying mechanisms we next identified endogenous microRNAs that target *Cd69* in DP thymocytes.

### A dual fluorescence reporter system identifies endogenous microRNAs that target the *Cd69* 3'UTR in DP thymocytes

The *Cd69* 3'UTR contains predicted sites for miR-181, miR-130 and miR-17/20 (http://www.targetscan.org) and there is firm experimental evidence for *Cd69* regulation by miR-181a, miR-130 and the miR-17-92 cluster (which encodes the microRNAs miR-17, -18, -19a, -19b, -20a, and -92 [34] in T lymphocytes [31–33].

**Fig 3:**
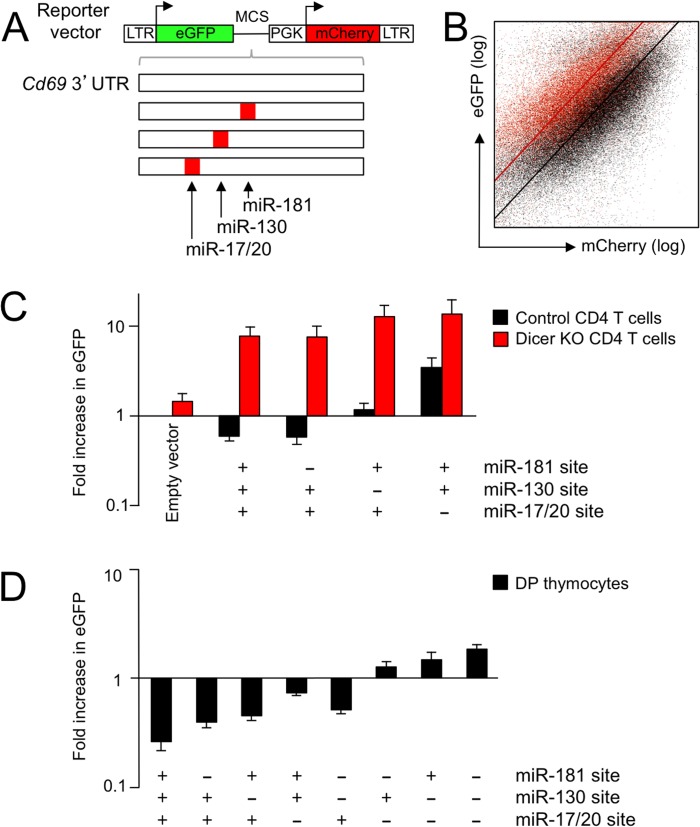
A dual fluorescence reporter system identifies endogenous microRNAs that target the *Cd69* 3'UTR in DP thymocytes. A) Dual fluorescence reporter based on retroviral vectors encoding eGFP followed by a multiple cloning site and mCherry for normalisation. Introduction of a 3'UTR containing relevant microRNA sites is predicted to downregulate eGFP expression relative to the mCherry control. The 842 nt 3’ UTR of *Cd69* contains predicted binding sites for miR-181, miR-130 and miR-17-20 starting at positions 255, 354 and 391, respectively, which were mutated alone and in combination. B) Representative log-log dot plot of mCherry and eGFP-*Cd69* 3'UTR expression by control (black) and Dicer-deficient mature CD4+ T cells isolated from lymph nodes (red). Fitted lines were used to calculate de-repression of eGFP. See S3A Fig. for additional data. C) Expression of eGFP obtained with empty vector and the indicated 3'UTR constructs in control (black) and Dicer-deficient mature CD4+ T cells isolated from lymph nodes (red, n = 4–14 per data point, mean ± SE). D) Expression of eGFP obtained with the indicated 3'UTR constructs in control DP thymocytes (n = 4–14 per data point, mean ± SE). See S3B Fig. for additional data.

To evaluate the impact of endogenous microRNAs on the expression of proteins linked to the 3'UTR of *Cd69* we developed a dual fluorescence reporter construct. The construct encodes the two fluorescent reporter proteins, eGFP and mCherry, under the control of separate retroviral long terminal repeat (LTR) and mouse *Pgk* promoters, as well as a cloning site 3’ of the eGFP transcript (Fig. 3A). In a manner similar to luciferase reporter constructs, 3’ UTRs of interest can be cloned into this site to measure their impact on the expression of GFP relative to mCherry. In contrast to heterologous reporter assays, however, this system allows to delineate the biological activity of endogenously expressed microRNAs after retroviral gene transfer of the reporter construct into primary cells. We characterised this dual fluorescence reporter system in mature CD4+ T cells that were isolated from lymph nodes and activated *in vitro* to render them receptive to retroviral gene transfer (S3A Fig.). To determine the impact of Dicer on the expression of eGFP linked to the 3'UTR of *Cd69* we transduced wild type and CD4Cre Dicer^lox/lox^ (Dicer-deficient) [35] CD4+ T cells with the reporter construct containing the entire *Cd69* 3'UTR (Fig. 3A, *Cd69* 3'UTR). Fig. 3B shows a representative dot plot of mCherry and eGFP-*Cd69* 3'UTR expression in control (black) versus Dicer-deficient CD4+ T cells (red). Compared to the empty control vector, wild type CD4+ T cells expressed eGFP-*Cd69* 3'UTR at a lower level and Dicer-deficient CD4+ T cells expressed eGFP-*Cd69* 3'UTR at a higher level (Fig. 3B, C), indicating that as well as repressive miRNA-binding sites, the CD69 3’ UTR may contain sequences that enhance expression. Mutation of the miR-181 site in the *Cd69* 3'UTR did not measurably affect the expression of eGFP in mature CD4+ T cells (Fig. 3C), which express only low levels of the developmentally regulated miR-181 [31]. However, deletion of the miR-130 and particularly the miR-17/20 site resulted in increased eGFP expression in wild type CD4+ T cells (Fig. 3C).

Next, thymocytes were transduced with *Cd69* 3'UTR reporter constructs and maintained for 24 hours in reaggregation thymic organ cultures until the expression of fluorescent reporters by CD4+ CD8+ DP thymocytes was recorded by flow cytometry. In contrast to mature CD4+ T cells, the miR-181 site affected eGFP-*Cd69* 3'UTR expression in CD4+ CD8+ DP thymocytes, which express maximal levels of the developmentally regulated miR-181 [31]. The predicted sites for miR-130 and miR-17/20 within the *Cd69* 3' UTR also affected eGFP reporter gene expression in DP thymocytes.

Taken together, these results show that eGFP-*Cd69* 3'UTR expression was Dicer-dependent in mature CD4+ T cells (Dicer-deficient thymocytes could not be used successfully for retroviral gene transduction and subsequent reaggregate organ cultures) and that the impact of predicted microRNA binding sites reflected the developmental regulation of microRNAs [31]. We focused our subsequent analysis on miR-181a and the miR-17-92 cluster.

### microRNA-181a controls cell-to-cell variability in CD69 expression

**Fig 4:**
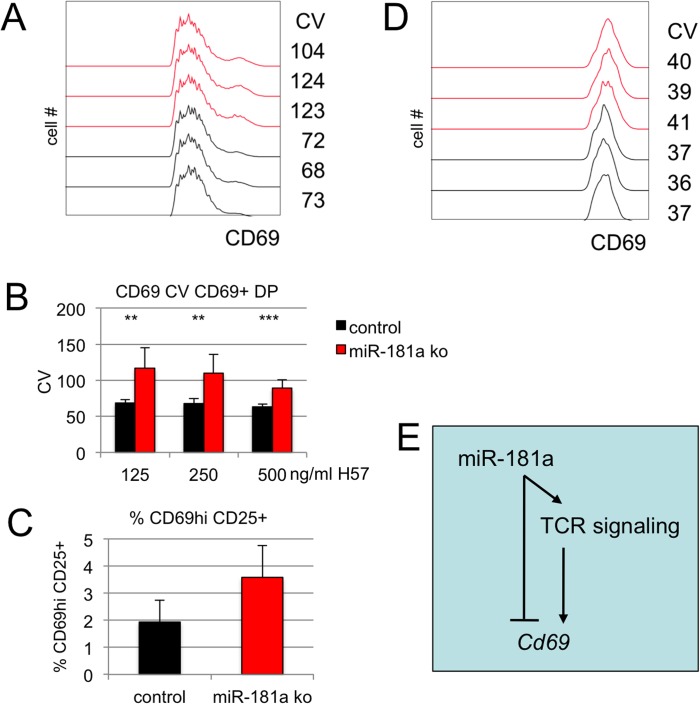
miR-181a controls cell-to-cell variation in CD69 expression. A) Histogram overlays of CD69 expression by control (black) and miR-181a-deficient (red) DP thymocytes activated for 18 hours with 125ng H57/ml. Histograms are gated on CD69+ cells. B) The CV of CD69 expression is higher in miR-181a-deficient than in control DP thymocytes (n = 7–8, ** p<0.005, *** p<0.0005). C) The frequency of CD69 hi CD25+ DP cells is higher in miR-181a-deficient than in control DP thymocytes (n = 12, P<10–5). D) The CV of CD69 expression in miR-181a-deficient and control CD69 hi CD25+ DP thymocytes is slightly higher than in control thymocytes. E) Model for the action of miR-181 upstream of TCR signaling and on *Cd69* mRNA.

To explore the influence of miR-181 on the CV of CD69 expression we analysed DP thymocytes deficient in *mir-181ab1*, which accounts for most of the miR-181a and -b copies in DP thymocytes [36]. Following activation, miR-181-deficient DP thymocytes showed increased mean CD69 expression (control = 245 ± 17, mean miR-181 ko = 278 ± 10, n = 26, P<10^–10^, 2-tailed T-test). Interestingly, CD69 expression in miR-181-deficient DP thymocytes also showed an increased CV (Fig. 4A) over a range of activation conditions (Fig. 4B). The increased CV was due mainly to a higher fraction of CD69^hi^ cells among miR-181-deficient DP thymocytes (Fig. 4C). The CV of CD69 expression within the CD69^hi^ subset was only mildly affected (Fig. 4D).

These results show that miR-181 is an important determinant of cell-to-cell variability in CD69 expression in activated DP thymocytes, and is required to restrict the fraction of CD69^hi^ DP cells. This is consistent with a role for miR-181 as a modulator of TCR signaling [36–38] (Fig. 4E).

### miR-17 and miR-20a form an incoherent positive feedforward loop with the target mRNA *Cd69*

miRNA expression responds to T-cell activation signals [34, 35, 39–45]. Many microRNAs are downregulated upon T-cell activation [40–43], but the expression of the miR-17-92 cluster is upregulated in activated mouse and human T cells [45]. Since the miR-17-92 cluster encodes microRNAs that target the *Cd69* 3'UTR, including miR-17 and miR-20a (Fig. 3), we investigated how the expression of miR-17 and miR-20a was affected by the activation of DP thymocytes.

**Fig 5:**
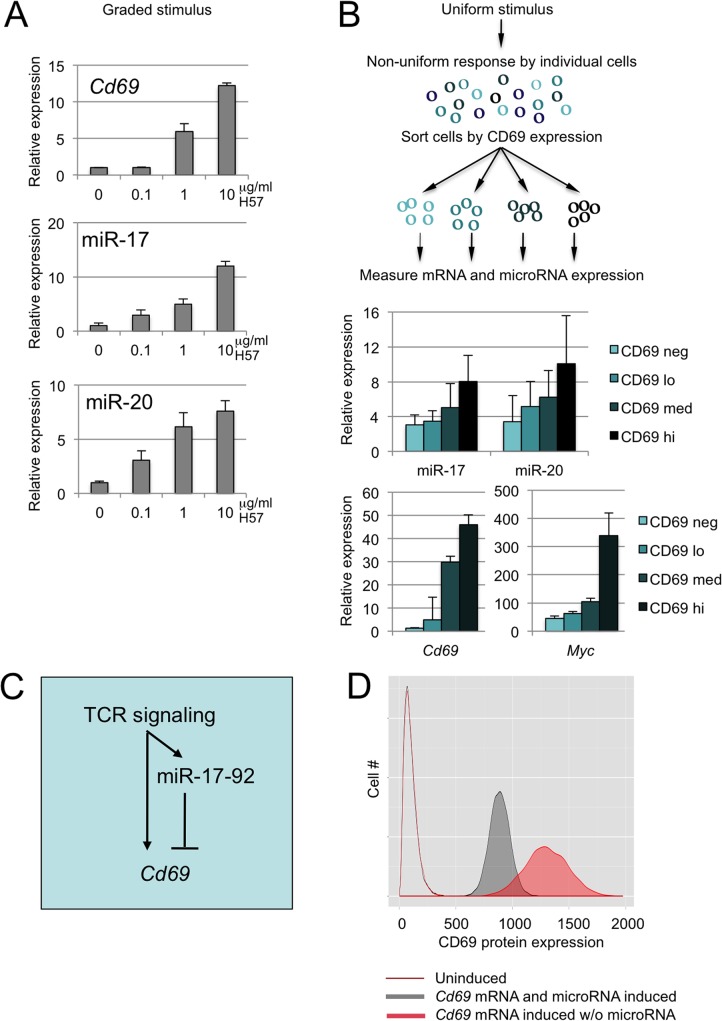
miR-17 and miR-20 form an incoherent positive feedback loop with the target mRNA *Cd69*. A) The strength of activation signals (0.1, 1, 10 μg/ml H57) determines the expression of *Cd69*, miR-17 and miR-20a (normalised to snRNA-135 and -202, n = 2–3, mean ± SE). B) Perceived signal strength varies among individual DP thymocytes and determines the expression of *Cd69*, miR-17 and miR-20a (microRNA expression is normalised to snoRNA-135 and snoRNA-202). At a fixed extracellular signal of 1u/ml H57, the fold-change in miR-17 and miR-20 relative to CD69 negative DP and normalised to snoRNA-135 was proportional to the expression of CD69 (n = 3, mean ± SD). C) The microRNA target mRNA *Cd69* and microRNAs of the miR-17-92 cluster are co-regulated in response to activation signals and form an incoherent feed-forward loop downstream of the TCR. D) Modeling CD69 protein expression with and without microRNA feed-forward regulation. The state of the system is described by five major variables: the number of mRNAs transcribed from the TF gene, the number of TF molecules, the number of miRNAs, the number of mRNAs and the number of target proteins. Of these variables we can estimate the number of mRNA copies and the number of microRNA copies. As detailed in the legend to S4 Fig., the number of *Cd69* mRNA copies was estimated as 0 in resting and 6 in activated cells, miR-17 and miR-20 were estimated as 6–12 copies per cell the resting state and 30–60 copies per cell after activation. Simulations of transcriptional networks were carried out using the Gillespie exact stochastic simulation algorithm, programmed and analysed using R based on a microRNA feed-forward model [8] to simulate CD69 protein expression in resting T cells (unfilled histogram), in a scenario where activation increases *Cd69* mRNA but the expression of miR-17 and miR-20a remained the same as in resting T cells (activated without microRNA FFL, filled red histogram), and in a scenario where activation increases both *Cd69* mRNA and miR-17 and miR-20a expression (activated with microRNA FFL, filled grey histogram). The plot represents 10,000 simulations. The model predicts that thymocyte activation with co-regulation of *Cd69* mRNA and miR-17/miR-20a reduces the mean (887 versus 1300) and the CV (10.2 versus 14.6) of CD69 expression compared to the regulation of *Cd69* mRNA alone (P<10^–4^). See S4 Fig. for details of the underlying circuitry, the parameters used, and a model based on microRNA effects on mRNA degradation [49].

We applied graded stimuli (0, 0.1, 1 and 10μg H57/ml) to induce a graded increase in *Cd69* mRNA expression (Fig. 5A). Interestingly, this graded increase in *Cd69* mRNA was accompanied by a proportional upregulation of miR-17 and miR-20a expression (Fig. 5A).

Next, we asked how miR-17 and miR-20a expression was related to the range of responses by individual cells to a uniform extracellular signal. When stimulated with a fixed concentration of TCR antibody, DP thymocytes expressed a range of CD69 protein, from undetectable to high (see Fig. 2A). We applied a uniform stimulus (1μg H57/ml) and sorted DP thymocytes that expressed no detectable CD69 protein (CD69 neg), low levels of CD69 (CD69 lo), intermediate levels of CD69 (CD69 med) or high levels of CD69 (CD69 hi). Increasing expression of CD69 protein correlated with increasing *Cd69* mRNA levels, and with incremental expression of miR-17 and miR-20a (Fig. 5B).

Hence, activation signals of increasing strength induce a proportional upregulation of the microRNAs miR-17 and miR-20a and the target mRNA *Cd69*. Furthermore, cells exposed to a uniform stimulus show a range of responses, and the induction of microRNAs and mRNA target is coordinated with the expression of the protein encoded by the target mRNA in individual cells. These findings suggest that miR-17, miR-20a and *Cd69* are co-regulated. Mechanistically, the transcription factor Myc provides a link between thymocyte activation and the coordinated regulation of *Cd69* and the miR-17-92 cluster. Myc expression is upregulated by signals that drive lymphocyte activation and mediates downstream transcriptional responses [46] (Fig. 5B). *Myc* and *Cd69* are induced by shared signaling pathways downstream of the TCR [47], and Myc directly activates transcription of the miR-17-92 cluster [48]. These data indicate that the microRNA target mRNA *Cd69* and microRNAs of the miR-17-92 cluster form an incoherent feed-forward loop in response to TCR signaling (Fig. 5C).

The coordinated regulation of miR-17, miR-20a and *Cd69* in response to TCR signaling provides a potential mechanism for restricting cell-to-cell variability of microRNA target gene expression. To explore this idea further we implemented computational models of noise regulation by microRNAs. In one model, a microRNA and target mRNA are induced together and the microRNAs inhibits the translation of the mRNA as part of an incoherent feedforward loop [8] (S4A Fig.). In an alternative model, a co-regulated pair of microRNA and mRNA interact to induce mRNA degradation [49] (S4B Fig.). Both models predict that microRNA feedforward regulation reduces the mean and the CV of target expression. To implement a more specific model of CD69 regulation we estimated the mRNA copy numbers for *Cd69* and the microRNA copy numbers for miR-17 and miR-20a in resting and activated T cells (see legend Fig. 5D). This model predicts that thymocyte activation results in mean CD69 expression of 887 with a CV of 10.2% when *Cd69* mRNA and miR-17/miR-20a are induced together (activated with microRNA FFL, filled grey histogram in Fig. 5D). In contrast, induction of *Cd69* mRNA without upregulation of miR-17/miR-20a results in a higher mean (1300) and increased CV (14.6%, activated without microRNA FFL, filled red histogram in Fig. 5D, P<10^–4^). This result is consistent with our experimental data where the mean and CV of activation-induced CD69 expression were significantly elevated in Dicer-deficient thymocytes: in the absence of a functional microRNA biogenesis pathway, the activation-driven increase in *Cd69* mRNA was not balanced by increased miR-17 and miR-20a expression.

## Discussion

microRNAs are essential for mammalian development [50] due to their diverse range of regulatory roles in gene expression. They facilitate developmental transitions by the reciprocal regulation of microRNAs and their targets in cell types derived from a common progenitor [23] and by participating in regulatory circuits with switch-like functions [5], buffer against environmental and genetic variation [2–5], limit intrinsic transcriptional noise (by allowing mRNA 'overproduction' and post-transcriptional removal of excess transcripts) [2, 51] and reduce extrinsic noise as part of FFLs [2, 7, 8, 10], as demonstrated in the current study for a mammalian developmental system.

The inducible expression of the established microRNA target *Cd69* [31–33] allowed us to explore molecular mechanisms by which microRNAs affect the cell-to-cell variability of target gene expression in thymocytes. miR-181 is a known modulator of TCR signal transduction [36–38] and our data show that the deletion of mir-181ab1 affected the CV of CD69 expression mainly by altering the proportion of thymocytes that expressed CD69 at high levels. The expression of miR-181a is downregulated as thymocytes mature [31] and in this way may account for developmentally regulated changes in the responsiveness of thymocytes to TCR signaling. Our data are consistent with this model and further suggest that developmental regulation of miR-181a reduces cell-to-cell variability of thymocyte responses to TCR signaling. A different mechanism applies to the regulation of CD69 by miR-17 and miR-20a, two microRNAs of the miR-17-92 cluster. Our data show that the expression of these microRNAs is induced together with *Cd69* mRNA in response to TCR signals, and that the expression of CD69 protein, *Cd69* mRNA and miR-17/miR20a is proportional in thymocytes. This co-regulation of microRNAs and target mRNA has the potential for feed-forward regulation. While the specific circuitry that places *Cd69* and miR-17-92 under the shared control of TCR signaling remains to be elucidated, *Myc* and *Cd69* are induced by shared signaling pathways downstream of the TCR [47], and Myc directly activates transcription of the miR-17-92 cluster [48].

Computational and experimental data suggest that FFLs can confer microRNA-mediated robustness of target gene expression by reducing noise that originates upstream of the transcription of the target mRNA itself [8, 10]. Modeling the impact of microRNA feedforward regulation either by translational inhibition or mRNA degradation predicted a reduction in the mean and CV of target expression [8, 49]. This was confirmed by modeling the experimentally estimated copy numbers of *Cd69* mRNA and miR-17 and miR-20a in resting and activated T cells. Of note, while all models captured the ability of microRNAs to reduce both the average expression and the CV of microRNA targets, they nevertheless overestimated the actual impact of microRNA-mediated feed-forward regulation. Neither model fully predicted the complexity of the data, specifically the experimentally observed skewing of expression at the top end of the expression spectrum. This indicates that the current models do not fully capture the integration of microRNAs into biological circuits and their impact on gene expression.

TCR signaling drives developmental decisions in thymocytes according to a specific set of rules: too little signal results in a failure to differentiate ('neglect'), too much signal results in activation-induced cell death ('negative selection') [24]. Intermediate signals induce thymocyte differentiation ('positive selection') towards CD4-expressing T helper and CD8-expressing cytotoxic T cells. The nature and strength of signals also directs differentiation towards specialized T cell subsets such as regulatory T cells (Treg cells) and natural killer T cells (NK-T). The functionality of CD4, CD8, Treg and NK-T cells depends on their TCR specificity and it is therefore critical that signal strength and lineage choice are appropriately matched [24, 52]. microRNAs are intimately involved in T cell lineage choices [26, 35, 36–38, 53–57]. The ability to mount predictable responses to extracellular signal is therefore as important for T cell development as it is for other developmental decisions and we speculate that the exploration of microRNA-mediated regulation of cell-to-cell variation in gene expression in other cell types will prove relevant for understanding normal development and disease.

## Materials and Methods

Mouse work was done according the UK Animals (Scientific Procedures) Act under the authority of project licences issued by the Home Office, UK. LckCre Dicer [26], CD4Cre Dicer [35] and mir-181ab1-deficient mice [36] have been described. Fixation and intracellular staining of thymocytes were done as described [39], Antibodies used were RM4-5 (anti-CD4), 53-6.7 (anti-CD8a), 53-5.8 (anti-CD8b), PC61 (anti-CD25), H1.2F3 (anti-CD69), IM7 (anti-CD44), E13-161.7 (anti-Ly6a), AF6-88.5 (anti-H2-K1), and 11-5.2 (anti-H2-Ak; Becton Dickinson) and cells were analysed and sorted on FACS Calibur, LSR II and FACS Aria instruments (Becton Dickinson, Oxford, UK).

Mature CD4+ T cells were activated with anti-CD3 and anti-CD28 for 24 hours, thymocytes were activated with the indicated concentrations of plate-bound T cell receptor beta antibody H57-597 and 2ug/ml of anti CD28 (37.51) for 18 hours.

Dual Fluorescence reporter constructs were based on pMSCVpuro plasmids (Clontech) and contained cDNAs for the fluorescent reporter proteins eGFP under the control of the retroviral LTR and mCherry under the control a separate *Pgk* promoter, as well as a cloning site in the 3’ UTR of eGFP for the introduction of 3’ UTRs. 3’ UTR fragments were cloned from lymphocyte cDNA and microRNA site mutations introduced by PCR. Retrovirus was produced and activated mature CD4+ T cells or newborn thymocytes were transduced by spin infection as described [58]. Cells were reaggregated with dissociated stromal cells from deoxyguanosine-treated embryonic thymi as described [59], recovered 24 hours later and reporter fluorescence was assessed by flow cytometry. To model the relationship between GFP and mCherry we used orthogonal linear regression, with the relative level of eGFP to mCherry calculated as the slope of the fitted line. These ratios of eGFP expression to cherry expression are normalised to the eGFP/mCherry ratio of the empty vector, to quantify the change in eGFP expression in experimental vectors compared to the empty vector. By comparing eGFP expression from control and Dicer-deficient cells the level of miRNA-dependent repression can also be observed.

RNA was extracted from three biological replicates of Dicer^lox/lox^ and Dicer^Δ/Δ^ DP thymocytes, and processed for Affymetrix Mouse Genome 430 2.1 array hybridisation as described [58]. Gene expression array data have been deposited at Geo under accession number GSE57511. Array data were analysed using dChip (http://www.dchip.org). Microarray probe sets were mapped to Refseq transcripts [60]. microRNA sequences were from miRBase [61]. 3' UTR nucleotide motifs were identified using miReduce [27].

Total RNA was isolated using RNAbee (Tel-Test, Friendswood, TX) and reverse transcribed. PCR reactions included 2x SYBR PCR Master Mix (Qiagen), 300nM primers and 2 μl of cDNA as a template in 50μl reaction volume. Cycle conditions were 94°C for 8 min, 40 cycles of 94°C for 30 sec, 55°C for 30 sec, 72°C for 1 min, followed by plate read. All primers amplified specific cDNAs with at least 95% efficiency. Data were normalized to the geometrical average of two housekeeping genes, using the CT method as outlined in the Applied Biosystems protocol for reverse transcriptase-PCR. Primer sequences were (5' to 3'):

Ywhaz fw CGTTGTAGGAGCCCGTAGGTCAT rev TCTGGTTGCGAAGCATTGGG

Ube fw AGGAGGCTGATGAAGGAGCTTGA rev TGGTTTGAATGGATACTCTGCTGGA

Computational modeling of microRNA effects on target gene expression was done as described [8, 49].

## Supporting Information

S1 Fig microRNA-retaining cells do not account for the increased cell-to-cell variation of protein expression in Dicer-deficient DP thymocytes. *Dicer* is deleted by Lck-Cre with 95–100% efficiency in DP thymocytes and Dicer-dependent microRNAs are reduced by ∼90% (Fig. 1A) [26]. We considered to what extent residual microRNA-retaining cells could contribute to cell-to-cell variation. If the residual microRNAs were evenly distributed across the population we would not expected this to affect the CV. The CV would be affected, however, if the residual microRNAs were concentrated in a subset of microRNA-retaining cells. This subset of cells would continue to repress microRNA targets and therefore show a lower mean than microRNA-deficient cells. Even if both populations individually had similar CVs, the resulting composite population would show a broader distribution. We addressed this 'worst case scenario' experimentally (A, B) and computationally (C). Mixing experiments with cells that were deliberately stained at 2-fold different intensities showed that 20% of microRNA-retaining cells would be required to significantly degrade the CV (A). Only 10% residual microRNA expression is observed experimentally (Fig. 1A) [26], which is not sufficient to explain the observed increase in CV. Furthermore, adding a subset of cells with lower mean expression to a population of cells with higher mean expression results in a skewed distribution of expression where more cells are below the peak channel than above the peak channel. This is the opposite of the experimentally observed distribution in Dicer-deficient DP thymocytes, which showed more cells above the peak channel than below the peak channel (B). Computational deconvolution ('unmixing') experiments indicated that 25% of microRNA-retaining cells would need to be removed from the fluorescence distribution of Dicer-deficient DP thymocytes to reduce their CV by 1% (C). Hence, the increased cell-to-cell variation of Dicer-deficient DP thymocytes was not explained by microRNA-retaining cells. A) Mixing populations with different means but similar CVs increases the CV of the resulting population. Experimental mixing of 20% of cells with a lower expression level (red) and 80% with a higher expression level (black) increases the CV and skews the resulting population (blue) away from a Gaussian distribution and towards the left. B) Experimentally determined staining profiles of control (grey) and Dicer-deficient DP thymocytes (red) were analysed for the distribution of cells above and below the peak channel (mean ± SD, n = 10). Note that the distribution of Dicer-deficient cells is skewed to the right. C) Effect of computationally removing hypothetical microRNA-retaining cells from the experimentally observed fluorescence distribution of Dicer-deficient cells. Plotted is the change in CV produced by computational removal of an increasing percentage of microRNA-retaining cells. Note that 25% of microRNA-retaining cells would need to be removed from the fluorescence distribution of Dicer-deficient DP thymocytes to reduce the CV by 1%. Error bars reflect simulations using 10 different sets of results.
(TIFF)

S2 Fig Graded activation signals induced a proportional increase of *Cd69* mRNA and CD69 protein. A) Graded activation signals induced a proportional increase of *Cd69* mRNA, representative of two similar experiments, see Fig. 5A and 5B for replicate determinations. B) Graded activation signals induced a proportional increase of CD69 protein with higher average CD69 expression in Dicer-deficient DP thymocytes. Shown is the mean CD69 expression by control and Dicer-deficient DP thymocytes activated as in Fig. 2. (n = 7–8 per data point, * P<0.05).
(TIFF)

S3 Fig Expression of dual fluorescence reporters in mature CD4+ T cells isolated from lymph nodes and in DP thymocytes. A) Dot plot of flow cytometry data from mature CD4+ T cells isolated from lymph nodes, activated for 24 hours and transduced with mCherry and eGFP-*Cd69* 3'UTR. Expression of eGFP and mCherry was measured by flow cytometry 24 hours after retroviral transduction. Cells in the upper right quadrant of the dot plot were used to calculate the impact of the 3'UTR on eGFP expression. B) Dot plot of flow cytometry data from DP thymocytes transduced with mCherry and eGFP-*Cd69* 3'UTR and subsequently maintained in reaggregate thymic organ culture. The expression of eGFP and mCherry was measured by flow cytometry 24 hours after retroviral transduction and cells in the upper right quadrant of the dot plot were used to calculate the impact of the 3'UTR on eGFP expression.
(TIFF)

S4 Fig Computational models of noise regulation by microRNAs. A) Schematic of a microRNA feedforward model in which miRNAs bind to mRNAs and inhibit mRNA translation (left), based on [8]. Output of 10,000 simulations of gene regulation with (black) and without (red) microRNA participation in translational repression (right. Parameters: rate of transcription factor (TF) transcription 0.06, rate of transcription factor and output mRNA degradation 0.006, rate of transcription factor translation 0.04, rate of transcription factor and output protein degradation 0.002, base rate of microRNA transcription 0.5, dissociation constant for transcription factor regulation of microRNA and target mRNA transcription 200, rate of microRNA degradation 0.006, rate of target mRNA transcription 0.8, base rate of mRNA translation 0.04, microRNA dissociation constant 60. All Hill coefficients are 2. In Fig. 5D this model was applied to predict the impact of microRNAs on CD69 protein expression using the following estimates of mRNA and microRNA copy numbers per cell. CD69 mRNA was barely detectable in resting cells and increased to ∼25.000 per 10^6^ copies of *B2M* in activated Jurkat T cells [62]. Based on the presence of ∼215 copies of *B2M* per cell [63], activated T cells contain ∼6 copies of *Cd69* mRNA per cell. miR-181a is present at 400 [36] to 800 [31] copies per DP thymocyte. Based on reported cloning frequencies (89884 miR-181a-1/2 per 10^6^ microRNAs in DP, 1465 miR-17 per 10^6^ microRNAs in DP thymocytes, and 1050 miR-20a per 10^6^ microRNAs in DP [64]. DP thymocytes contain ∼6–12 copies of miR-17 and miR-20a per cell, and our quantitative PCR data show that this number increases by 5–10-fold in response to TCR signaling. B) Schematic of a microRNA feedforward model in which microRNAs bind to mRNAs and enhance mRNA degradation (left), based on [49]. Output of 10,000 simulations of gene regulation with (black) and without (red) microRNA participation in mRNA degradation (right). Rate constants are as in C), but without translational repression. The rate of output protein translation is 0.04. The rate constant of microRNA-mRNA complex formation and dissociation is 0.0001, and the rate constant for mRNA degradation in complex is 0.02.
(TIFF)

S1 Table Mean and CV of flow cytometry data presented in Fig. 2B-F. (XLSX)
