# Supplementary figures and images for: microRNAs Regulate Cell-to-Cell Variability of Endogenous Target Gene Expression in Developing Mouse Thymocytes
Source: PLoS Genet. 2015 Feb 25;11(2):e1005020. doi: 10.1371/journal.pgen.1005020 (PMC4340958; doi:10.1371/journal.pgen.1005020)

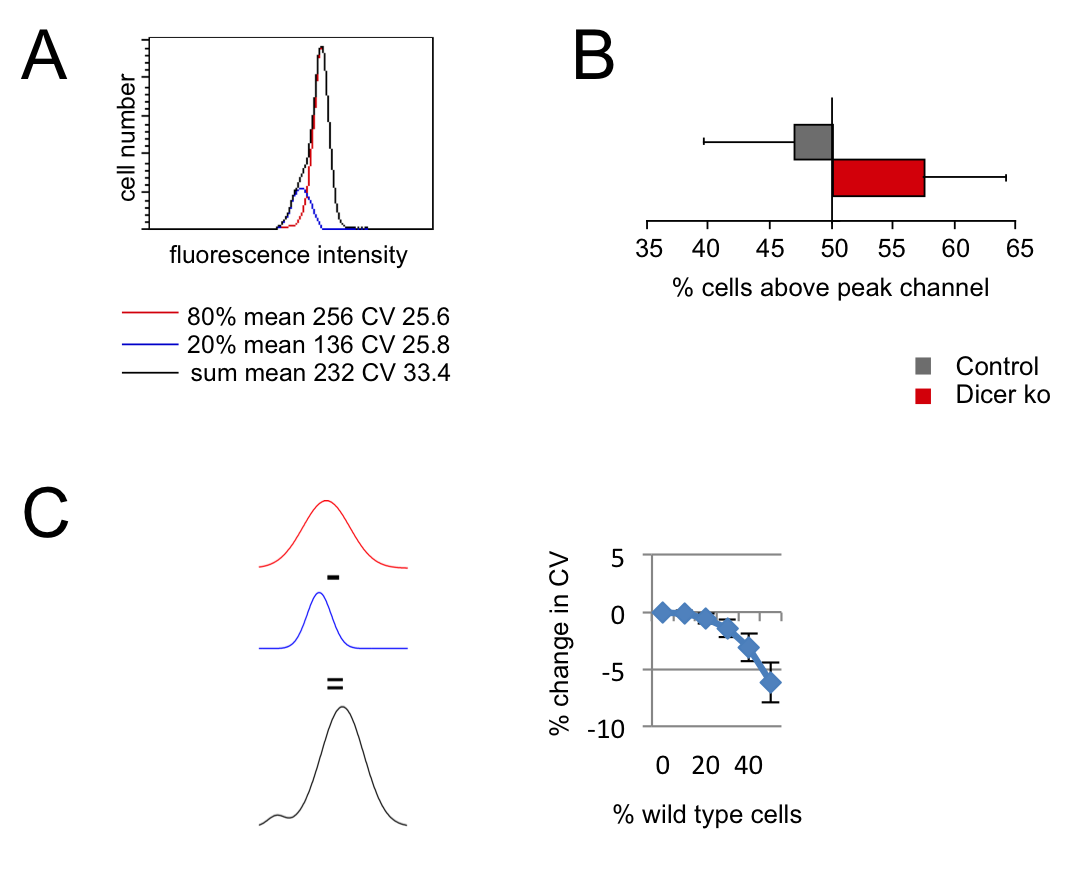

Supplement: S1 Fig — Dicer is deleted by Lck-Cre with 95–100% efficiency in DP thymocytes and Dicer-dependent microRNAs are reduced by ∼90% (Fig. 1A) [26]. We considered to what extent residual microRNA-retaining cells could contribute to cell-to-cell variation. If the residual microRNAs were evenly distributed across the population we would not expected this to affect the CV. The CV would be affected, however, if the residual microRNAs were concentrated in a subset of microRNA-retaining cells. This subset of cells would continue to repress microRNA targets and therefore show a lower mean than microRNA-deficient cells. Even if both populations individually had similar CVs, the resulting composite population would show a broader distribution. We addressed this 'worst case scenario' experimentally (A, B) and computationally (C). Mixing experiments with cells that were deliberately stained at 2-fold different intensities showed that 20% of microRNA-retaining cells would be required to significantly degrade the CV (A). Only 10% residual microRNA expression is observed experimentally (Fig. 1A) [26], which is not sufficient to explain the observed increase in CV. Furthermore, adding a subset of cells with lower mean expression to a population of cells with higher mean expression results in a skewed distribution of expression where more cells are below the peak channel than above the peak channel. This is the opposite of the experimentally observed distribution in Dicer-deficient DP thymocytes, which showed more cells above the peak channel than below the peak channel (B). Computational deconvolution ('unmixing') experiments indicated that 25% of microRNA-retaining cells would need to be removed from the fluorescence distribution of Dicer-deficient DP thymocytes to reduce their CV by 1% (C). Hence, the increased cell-to-cell variation of Dicer-deficient DP thymocytes was not explained by microRNA-retaining cells. A) Mixing populations with different means but similar CVs increases the CV of th [file pgen.1005020.s001.tiff]

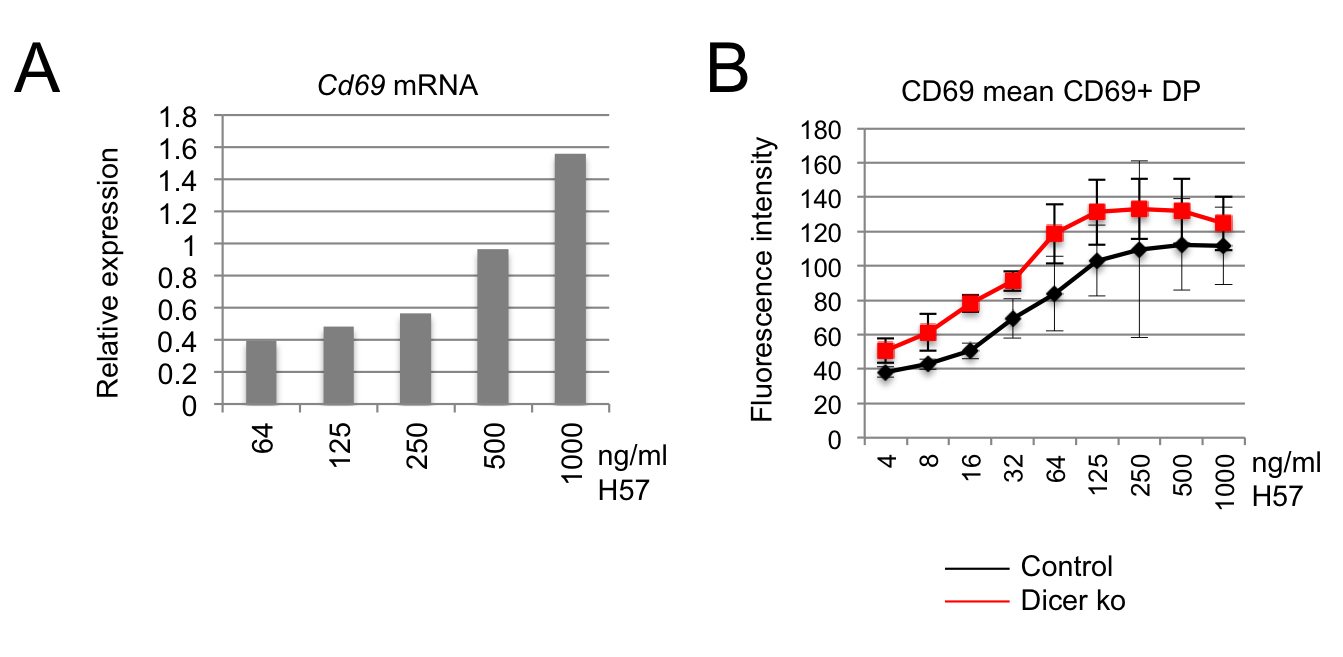

Supplement: S2 Fig — A) Graded activation signals induced a proportional increase of Cd69 mRNA, representative of two similar experiments, see Fig. 5A and 5B for replicate determinations. B) Graded activation signals induced a proportional increase of CD69 protein with higher average CD69 expression in Dicer-deficient DP thymocytes. Shown is the mean CD69 expression by control and Dicer-deficient DP thymocytes activated as in Fig. 2. (n = 7–8 per data point, * P<0.05). (TIFF) [file pgen.1005020.s002.tiff]

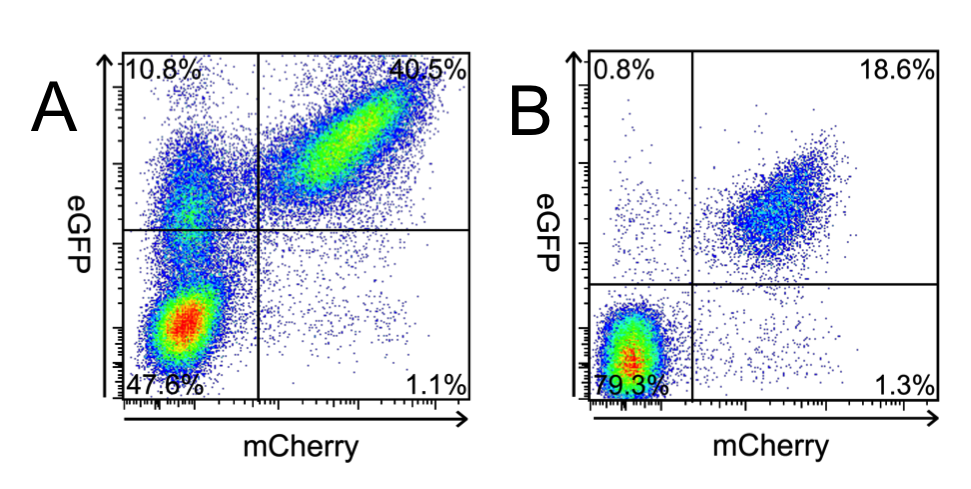

Supplement: S3 Fig — A) Dot plot of flow cytometry data from mature CD4+ T cells isolated from lymph nodes, activated for 24 hours and transduced with mCherry and eGFP-Cd69 3'UTR. Expression of eGFP and mCherry was measured by flow cytometry 24 hours after retroviral transduction. Cells in the upper right quadrant of the dot plot were used to calculate the impact of the 3'UTR on eGFP expression. B) Dot plot of flow cytometry data from DP thymocytes transduced with mCherry and eGFP-Cd69 3'UTR and subsequently maintained in reaggregate thymic organ culture. The expression of eGFP and mCherry was measured by flow cytometry 24 hours after retroviral transduction and cells in the upper right quadrant of the dot plot were used to calculate the impact of the 3'UTR on eGFP expression. (TIFF) [file pgen.1005020.s003.tiff]

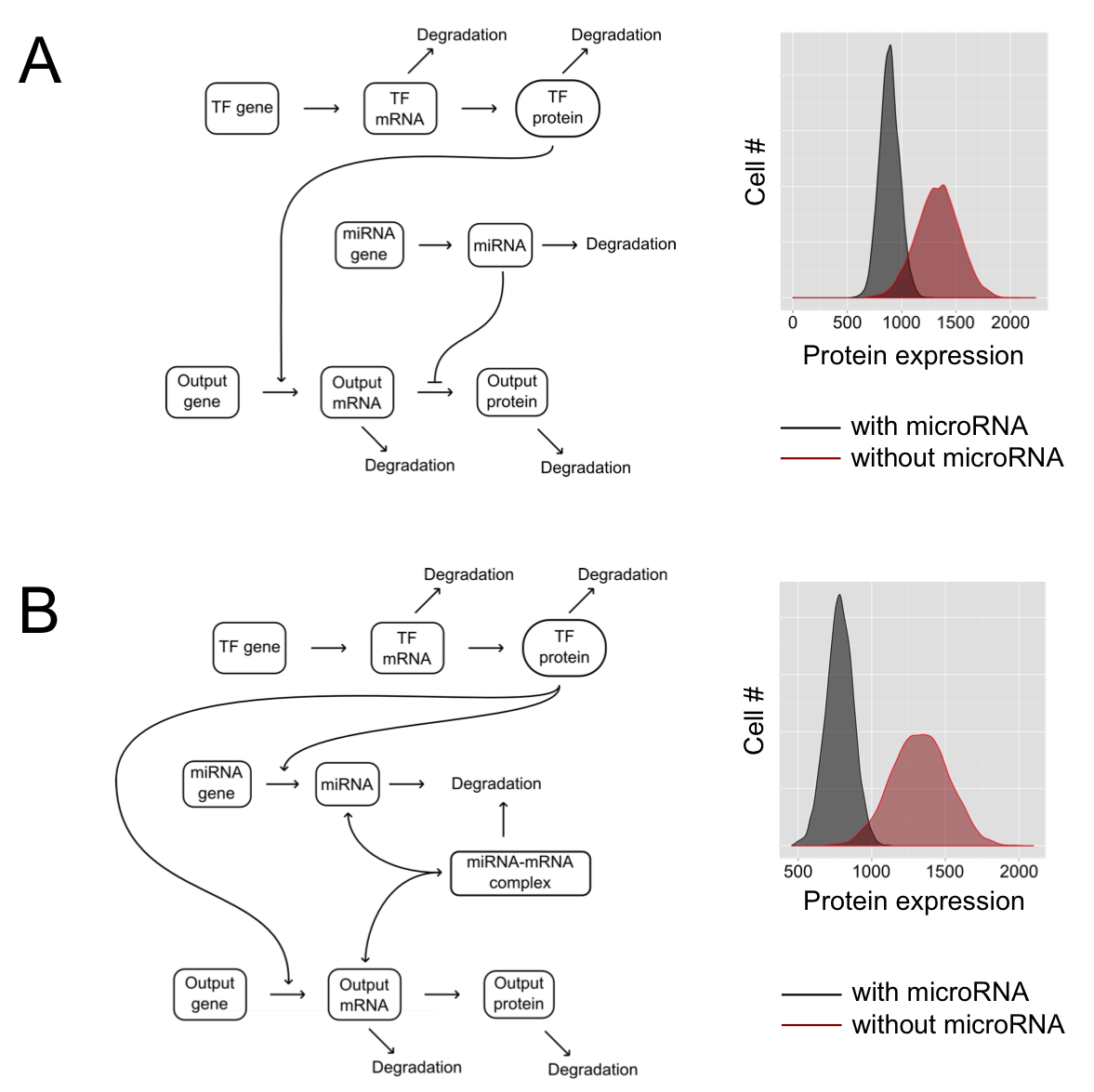

Supplement: S4 Fig — A) Schematic of a microRNA feedforward model in which miRNAs bind to mRNAs and inhibit mRNA translation (left), based on [8]. Output of 10,000 simulations of gene regulation with (black) and without (red) microRNA participation in translational repression (right. Parameters: rate of transcription factor (TF) transcription 0.06, rate of transcription factor and output mRNA degradation 0.006, rate of transcription factor translation 0.04, rate of transcription factor and output protein degradation 0.002, base rate of microRNA transcription 0.5, dissociation constant for transcription factor regulation of microRNA and target mRNA transcription 200, rate of microRNA degradation 0.006, rate of target mRNA transcription 0.8, base rate of mRNA translation 0.04, microRNA dissociation constant 60. All Hill coefficients are 2. In Fig. 5D this model was applied to predict the impact of microRNAs on CD69 protein expression using the following estimates of mRNA and microRNA copy numbers per cell. CD69 mRNA was barely detectable in resting cells and increased to ∼25.000 per 106 copies of B2M in activated Jurkat T cells [62]. Based on the presence of ∼215 copies of B2M per cell [63], activated T cells contain ∼6 copies of Cd69 mRNA per cell. miR-181a is present at 400 [36] to 800 [31] copies per DP thymocyte. Based on reported cloning frequencies (89884 miR-181a-1/2 per 106 microRNAs in DP, 1465 miR-17 per 106 microRNAs in DP thymocytes, and 1050 miR-20a per 106 microRNAs in DP [64]. DP thymocytes contain ∼6–12 copies of miR-17 and miR-20a per cell, and our quantitative PCR data show that this number increases by 5–10-fold in response to TCR signaling. B) Schematic of a microRNA feedforward model in which microRNAs bind to mRNAs and enhance mRNA degradation (left), based on [49]. Output of 10,000 simulations of gene regulation with (black) and without (red) microRNA participation in mRNA degradation (right). Rate constants are as in C), but without translational repression. The ra [file pgen.1005020.s004.tiff]
